# Error in Results

**DOI:** 10.1001/jamanetworkopen.2024.54628

**Published:** 2024-12-10

In the Original Investigation titled “Modeling the Population Equity of Alzheimer Disease Treatments in the US,”^1^ published October 31, 2024, it was reported that $29.1 trillion in incremental costs of treatment were observed; however, this dollar amount should have been reported as $29.1 billion. This article has been corrected.
